# Low kidney uptake of GLP-1R-targeting, beta cell-specific PET tracer, ^18^F-labeled [Nle^14^,Lys^40^]exendin-4 analog, shows promise for clinical imaging

**DOI:** 10.1186/s13550-016-0243-2

**Published:** 2016-12-13

**Authors:** Kirsi Mikkola, Cheng-Bin Yim, Paula Lehtiniemi, Saila Kauhanen, Miikka Tarkia, Tuula Tolvanen, Pirjo Nuutila, Olof Solin

**Affiliations:** 1 Turku PET Centre, University of Turku, Turku, Finland; 2 MediCity Research Laboratory, University of Turku, Turku, Finland; 3 Turku PET Centre, Åbo Akademi University, Turku, Finland; 4 Division of Digestive Surgery and Urology, Turku University Hospital, Turku, Finland; 5 Department of Pharmacology, University of Helsinki, Helsinki, Finland; 6 Department of Medical Physics, Turku University Hospital, Turku, Finland; 7 Department of Endocrinology, Turku University Hospital, Turku, Finland; 8 Accelerator Laboratory, Åbo Akademi University, Turku, Finland; 9 Department of Chemistry, University of Turku, Turku, Finland

**Keywords:** Pancreas, β cell, Exendin, PET, ^18^F

## Abstract

**Background:**

Several radiometal-labeled, exendin-based tracers that target glucagon-like peptide-1 receptors (GLP-1R) have been intensively explored for β cell imaging. The main obstacle has been the high uptake of tracer in the kidneys. This study aimed to develop a novel GLP1-R-specific tracer, with fluorine-18 attached to exendin-4, to label β cells for clinical imaging with PET (positron emission tomography). We hypothesized that this tracer would undergo reduced kidney uptake. ^18^F-labeled [Nle^14^,Lys^40^]exendin-4 analog ([^18^F]exendin-4) was produced via Cu-catalyzed click chemistry. The biodistribution of [^18^F]exendin-4 was assessed with ex vivo organ γ-counting and in vivo PET imaging. We also tested the in vivo stability of the radiotracer. The localization of ^18^F radioactivity in rat and human pancreatic tissue sections was investigated with autoradiography. Receptor specificity was assessed with unlabeled exendin-3. Islet labeling was confirmed with immunohistochemistry. The doses of radiation in humans were estimated based on biodistribution results in rats.

**Results:**

[^18^F]exendin-4 was synthesized with high yield and high specific activity. Results showed specific, sustained [^18^F]exendin-4 uptake in pancreatic islets. In contrast to previous studies that tested radiometal-labeled exendin-based tracers, we observed rapid renal clearance of [^18^F]exendin-4.

**Conclusions:**

[^18^F]exendin-4 showed promise as a tracer for clinical imaging of pancreatic β cells, due to its high specific uptake in native β cells and its concomitant low kidney radioactivity uptake.

## Background

The determination of β cell mass is a challenge, because the target is a diffuse collection of cell clusters, highly dispersed throughout the pancreas. Indeed, the β cell mass constitutes less than 2% of the total pancreas mass, and this mass is likely to decrease during the course of diabetes. The ongoing development of therapeutic approaches to diabetes, whether based on pharmacology or islet transplantation, also calls for the development of methods for longitudinal in vivo assessments of β cell mass and function. There is an unmet need for an imaging agent that offers potential for clinical trial applications.

Glucagon-like peptide-1 (GLP-1) is secreted from the intestine in response to elevated blood glucose levels. GLP-1 binds to specific receptors on pancreatic islets to stimulate insulin secretion. Exendin-4 is a long-acting agonist of the glucagon-like peptide-1 receptor (GLP-1R); it mimics the actions of short-lived GLP-1 (plasma half-life <2 min). Several efforts have been made to label pancreatic β cells with exendin-based tracers that carry radiometals, such as ^99m^Tc and ^111^In for SPECT imaging [1, 2] and ^64^Cu and ^68^Ga for PET imaging [3]. However, high radioactivity concentrations were observed in the kidney. During imaging, this high radioactivity compromises tissue visualization in the kidney region, limits quantitative imaging, and reduces diagnostic accuracy. Because the kidney is a radiosensitive organ, the radiation burden might rapidly become unacceptably high with long-lived radioisotopes [4]. Some success has been achieved in efforts to reduce the renal radioactivity of various radiolabeled peptides. For example, some studies co-administered amifostine, albumin derivatives, poly-glutamic acid, or Gelofusine [5–7] to limit renal uptake; and others used cleavable linkers, to allow excretion of radioactive metabolites into the urine [8, 9].

Most peptides are excreted via the kidneys, but studies have shown that persistent retention is characteristic for radiometal-labeled exendin derivatives [10]. As an alternative, preclinical studies were conducted with ^18^F-labeled exendin-4; they showed high, sustained tracer uptake in INS-1 tumor cells and xenograft models [11]. However, importantly, after initially high radioactivity levels in the kidneys, clearance from the kidneys was rapid, compared to radiometal-labeled analogs [12, 13]. Wu et al. [14] observed specific uptake of [^18^F]TTCO-Cys^40^-exendin-4 in islets transplanted into NOD/SCID mice but limited kidney retention. These results suggested a need for further development of [^18^F]exendin-4 analogs for clinical applications.

The present study aimed to develop a ^18^F-labeled [Nle^14^,Lys^40^]exendin-4 tracer ([^18^F]exendin-4) via copper-catalyzed click chemistry. This tracer could be clinically useful for imaging pancreatic β cells to calculate mass. To date, no optimal tracers are available for this application. The vesicular monoamine transporter type II (VMAT2) has been suggested as a target to calculate β cell mass, but so far issues with this approach remain controversial [15–17].

## Methods

### Precursors and radiochemistry

**Fig. 1:**
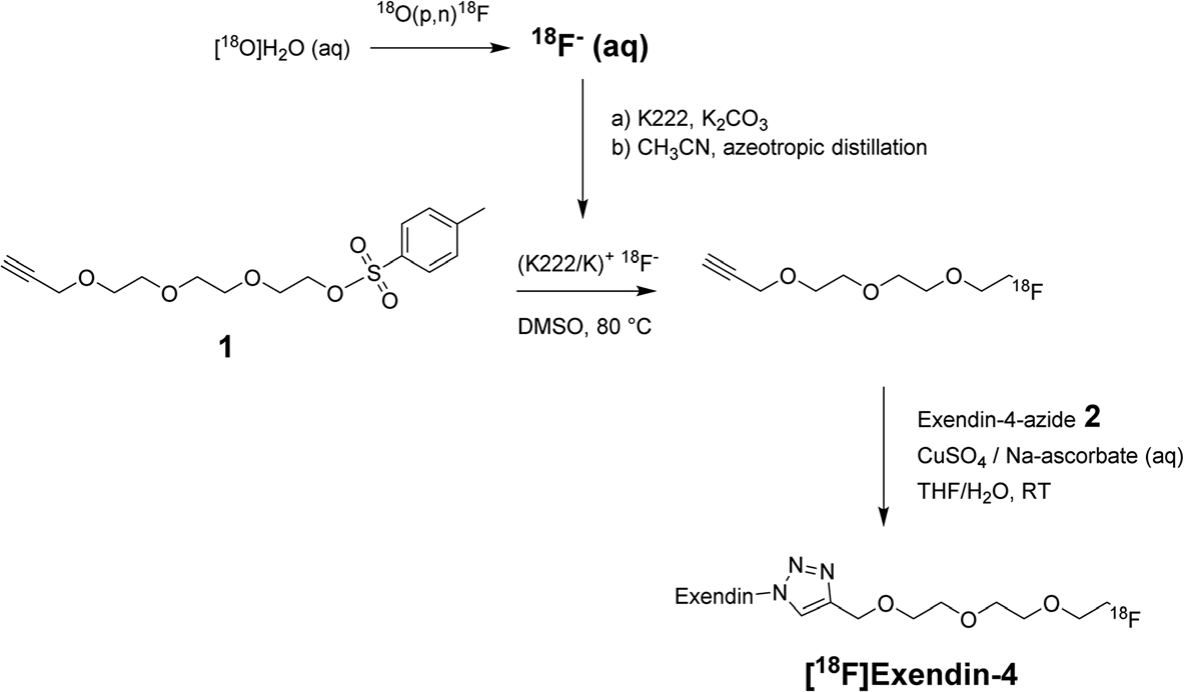
Production of [^18^F]exendin-4. ^18^F-fluoride (aqueous) and tosylate (**1**) were combined in a nucleophilic reaction for ^18^F-prelabeling; the product, ^18^F-fluoro-propargyl-triethylene glycol, was attached to exendin-4-azide (**2**) via Cu(I)-catalyzed alkyne/azide cycloaddition to produce radiofluorinated exendin-4

Tosyl-propargyl-triethylene glycol **1** was purchased from Huayi Isotope Co. (Toronto, Canada) (Fig. 1). Exendin-4-azide **2** was purchased from Peptide Specialty Laboratories GmbH (Heidelberg, Germany). Its amino acid sequence is HGEGTFTSDLSKQBEEEAVRLFIEWLKNGGPSSGAPPPSZ (B = norleucine and Z = azido-lysine-amide). [^19^F]Exendin-4 was synthesized from [^19^F]fluoro-propargyl-triethylene glycol (ABX GmbH, Radeberg, Germany) and peptidyl azide **2** following Cu(I)-catalyzed alkyne/azide cycloaddition in tetrahydrofuran (THF)/water, using aqueous solution of copper sulfate/sodium ascorbate. [^19^F]Exendin-4 was isolated by high-performance liquid chromatography (HPLC) (Jupiter C_12_ semi-preparative column, Phenomenex, Torrance, CA, USA).

Aqueous [^18^F]fluoride was produced by proton bombardment of ^18^O-enriched water as reported previously [18]. Synthesis started by allowing tosylate **1** to react with a [^18^F]fluoride-Kryptofix complex in anhydrous DMSO (Fig. 1). After purification by HPLC, the collected fraction was concentrated on an Oasis HLB cartridge (Waters, Milford, MA, USA). After elution of the cartridge with THF, the obtained [^18^F]fluoro-propargyl-triethylene glycol was allowed to react with peptidyl azide **2** in the presence of pre-activated copper sulfate/sodium ascorbate for 10 min at room temperature. Subsequently, the reaction mixture was diluted in water and purified by HPLC. The collected fraction was retained on a Sep-Pak C8 cartridge (Waters) and formulated in ethanol/saline. Radiochemical analysis of ^18^F-labeled [Nle^14^,Lys^40^]exendin-4 ([^18^F]exendin-4) was performed with HPLC and thin layer chromatography (TLC). The reaction details and a full characterization of the tracer will be reported elsewhere.

### Experimental animals and tissue samples of human pancreas

Sprague–Dawley rats (285 ± 30 g, *N* = 43) were obtained from the Central Animal Laboratory, University of Turku. Finnish landrace pigs (33 ± 3 kg, *N* = 3) were obtained from a local farmer. The animal experiments were approved by the animal experiment board of the University of Turku and by the State Provincial Office of Southern Finland (ESAVI/3899/04.10.07/2013, ESAVI/823/04.10.07/2013). Human pancreatic tissue was obtained from one subject (woman, 36 years old), who had undergone a pancreatic resection (pancreatic mucinous neoplasm) at Turku University Hospital (Turku, Finland). Approval for the use of human pancreatic tissue was obtained from the Ethics Committee of the Hospital District of Southwest Finland (ETMK 42/180/2008). The subject provided written informed consent before participating in the study.

### Small-animal PET imaging

The in vivo kinetics of [^18^F]exendin-4 were evaluated with the Inveon Multimodality PET/CT scanner (Siemens, Knoxville, TN, USA). Rats were anesthetized with isoflurane/O_2_ and injected intravenously (i.v.) with [^18^F]exendin-4 (radioactivity 23 ± 5 MBq/kg, mass 2 ± 1 μg/kg). PET scans were acquired for up to 6 h (dynamic 0–60 min; static 210–240 min and 330–360 min; *N* = 2–5 rats/scan). The data were reconstructed with the ordered subset expectation maximization OSEM2D algorithm. Analyses were performed with Inveon Research Workplace 3 software (Siemens), after defining the volumes of interest (VOIs) on selected tissues.

### Ex vivo biodistribution

Rats were anesthetized with isoflurane/O_2_ and injected (i.v.) with [^18^F]exendin-4 (radioactivity 23 ± 5 MBq/kg, mass 2 ± 1 μg/kg). After injection, animals were allowed to recover from the anesthesia (except animal sacrificed 15 min p.i.). We further investigated tracer distribution in the brain by injecting one rat with a highly radioactive dose (50 MBq) of [^18^F]exendin-4. Animals were sacrificed at 15, 30, 60, 120, 240, and 360 min (*N* = 2–11 rats/time point). Blood was extracted, and selected organs were dissected and weighed. Tissue radioactivity was measured with a γ-counter (Wizard, PerkinElmer, Turku, Finland). Tracer specificity was tested by co-injecting radiolabeled exendin-4 with an excess of cold exendin-3 (3 mg/kg) (Peptide Specialty Laboratories GmbH). Measurements were corrected for radionuclide decay, and results were reported as the percentage of the injected dose of radioactivity per gram of tissue (%ID/g).

### Ex vivo autoradiography

The kidney, pancreas, and brain were sectioned and exposed to imaging plates (Fuji BAS-TR2025; Fuji Photo Film Co., Tokyo, Japan) for approximately 4 h. Imaging plates were scanned with a BAS-5000 scanner (Fuji Photo Film Co.) and the images were analyzed with AIDA 4.5 software (Raytest, Isotopenmessgeräte, Straubenhardt, Germany). Three pancreatic sections per rat were analyzed (*N* = 2–8 rats/time point). We determined the mean density values (photostimulated luminescence per square millimeter, PSL/mm^2^) of ten prominently labeled islets and of the exocrine tissue and corrected for background noise. Finally, the islet-to-exocrine tissue ratio was calculated. This was done in order to be able to demonstrate the accumulation of radioactivity into the islets as a function of time and to be able to compare the islet-to-exocrine tissue ratios between animals of different time points. The pancreatic sections used for autoradiography were then stained with insulin antibody (1’ mouse-anti-insulin, Nordic Biosite, Täby, Sweden, 2’ MAHC1 mouse probe, Biocare Medical, Concord, CA, USA), counterstained with hematoxylin, and imaged with a Slide Scanner (Pannoramic 250 F, 3DHistech, Budapest, Hungary).

### Metabolite assay

The metabolic stability of [^18^F]exendin-4 was evaluated by radio-HPLC, as reported previously [3]. Briefly, samples were collected at 5, 15, 60, and 120 min post injection (p.i.; *N* = 1–3 samples/time point). Plasma and urine proteins were precipitated with equivalent volumes of acetonitrile and removed by centrifugation. Samples of the pancreas, kidney, and duodenum (pyloric sphincter) were homogenized in acetonitrile, purified by centrifugation, and the supernatants were analyzed with radio-HPLC.

In addition, the stability of [^18^F]exendin-4 was determined in pig plasma. Prior to blood sampling, animals were anesthetized (midazolam 1 mg/kg, xylazine 4 mg/kg intramuscular), connected to a respirator, and ventilated mechanically. Anesthesia was maintained with an i.v. infusion of propofol (10–20 mg/kg/h) and fentanyl (4–8 μg/kg/h). Vital signs were monitored throughout the study. Pigs were injected (radioactivity 1.2 ± 0.2 MBq/kg, mass 0.14 ± 0.04 μg/kg) and blood was sampled at 5, 15, 30, 60, and 180 min p.i. (*N* = 3 samples/time point). Plasma was treated and analyzed as described above.

### In vitro [^18^F]exendin-4 binding in human and rat pancreas and rat brain

We acquired a piece of human pancreas that was frozen immediately after surgery. We dissected a rat pancreas and brain and froze them immediately after dissection. Prior to incubations with ligand, the tissue sections of human and rat pancreas and GLP-1R-rich areas from rat brain [19] were incubated for 30 min with 1% BSA in PBS (pH 7.4). Then, sections were incubated for 60 min with various concentrations of [^18^F]exendin-4 (range 2.5–20 nM) in vitro. To investigate tracer specificity, sections were incubated for 30 min with an excess of cold exendin-3 prior to adding [^18^F]exendin-4. Sections were then washed and exposed to imaging plates, as described above (ex vivo autoradiography). Next, sections were stained and processed, as described above.

### Estimation of radiation doses for humans

Absorbed doses were calculated with OLINDA/EXM1.0 software [20, 21], which includes radionuclide information and a selection of human body phantoms. Rat ex vivo biodistributions were estimated by integrating the area under the time–activity curves. The obtained residence times were converted into corresponding human values by multiplying with organ-specific factors that scaled organs to body weights, as follows: (*W*_TB,rat_/*W*_Organ,rat_) × (*W*_Organ,human_/*W*_TB,human_), where *W*_TB,rat_ and *W*_TB,human_ are the total body weights of rats and humans (70-kg male), respectively; and *W*_Organ,rat_ and *W*_Organ,human_ are the organ weights of rats and humans (70-kg male), respectively.

### Statistical analyses

Results are reported as the mean ± SD. The two-tailed, unpaired Student’s *t* test and one-way ANOVA were used for determining statistical differences between groups (GraphPad Prism 5.01, GraphPad Software, San Diego, CA, USA). Linear regression analysis was used to assess the relationship between the islet-to-exocrine tissue ratio and time. Values of *P* < 0.05 were considered statistically significant.

## Results

### Radiochemistry

The synthesis of [^18^F]exendin-4 is depicted in Fig. 1. The copper(I)-catalyzed reaction between exendin-4-azide **2** and [^18^F]fluoro-propargyl-triethylene glycol proceeded rapidly and efficiently under mild reaction conditions. Technical details of the synthesis optimization studies will be reported elsewhere. The absence of major radioactive by-products ensured neat purification using HPLC. Total synthesis time was approximately 1.5 h. The average isolated yields of [^18^F]exendin-4 after HPLC purification ranged from 14 to 338 MBq (at the end of synthesis), when the starting amount of aqueous [^18^F]fluoride was 2.5–18 GBq. The specific activity for [^18^F]exendin-4 was 12–323 MBq/nmol at end of synthesis, with a radiochemical purity exceeding 90%. Identity of [^18^F]exendin-4 was confirmed by HPLC comparison test with the cold standard.

### Ex vivo biodistribution and PET imaging

**Fig. 2:**
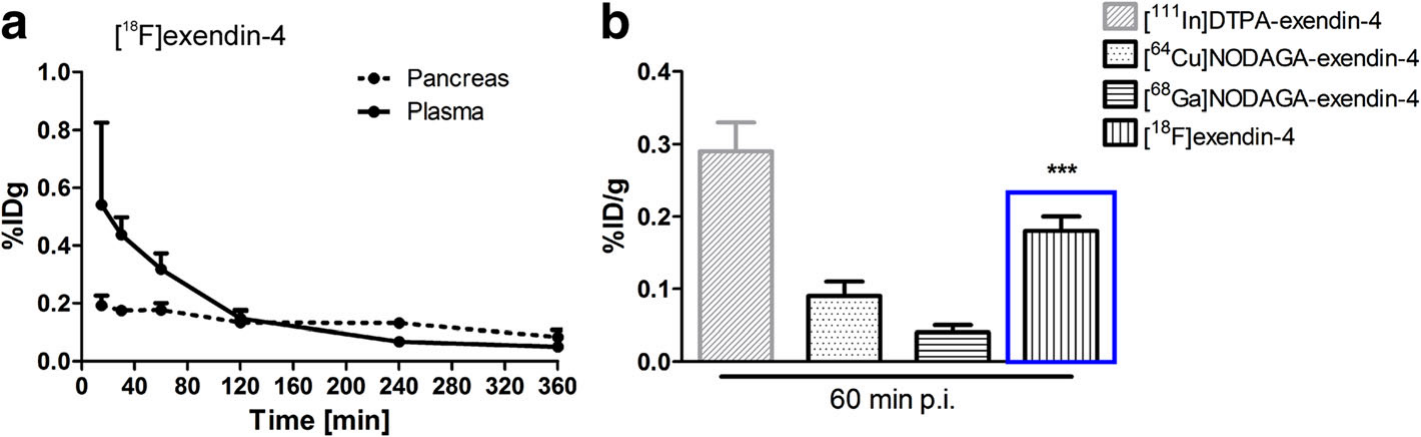
Ex vivo measurements of the accumulation of exendin-based tracers in rat pancreas. **a** In the pancreas (*dashed line*), the radioactivity uptake of [^18^F]exendin-4 was rather constant over the course of the study (*N* = 2–11 rats/time point). **b** The [^18^F]exendin-4 uptake (highlighted with a *blue box*) in the pancreas was significantly higher (*P* = 0.0001) than uptake of the ^64^Cu- and ^68^Ga-labeled PET tracer counterparts (one-way ANOVA) [3]. Conversely, the uptake of the [^111^In]exendin-4 SPECT tracer (*grey*) was higher than uptake of the PET tracers [2]

**Fig. 3:**
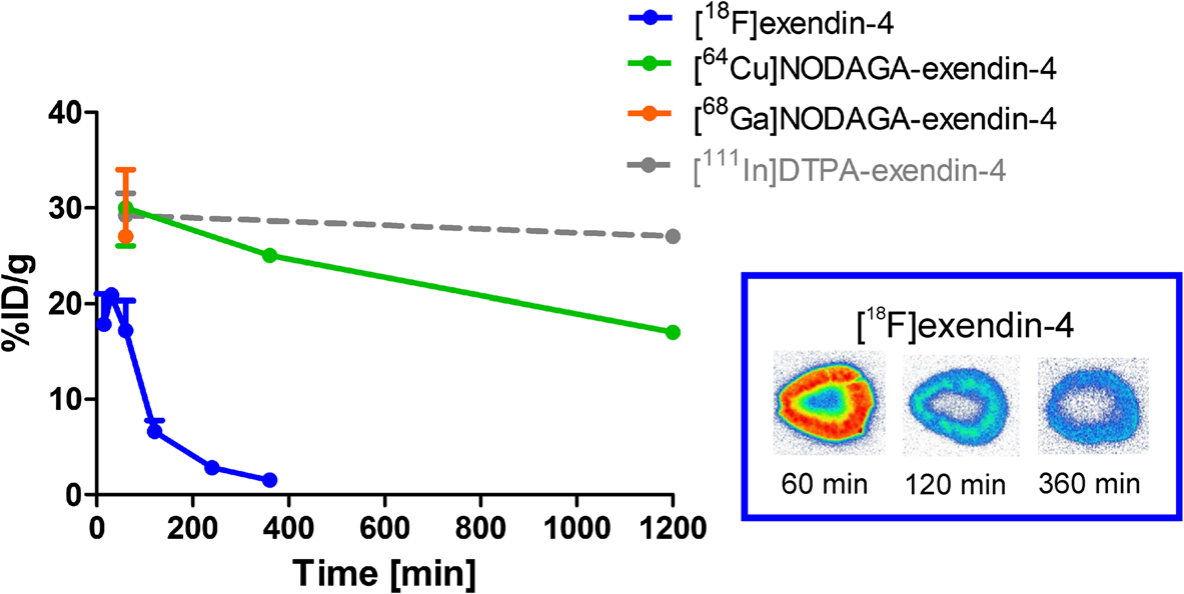
Ex vivo measurements of accumulation of exendin-based PET and SPECT tracers in rat kidney. The renal clearance of [^18^F]exendin-4 (*blue*) was rapid, and the radioactivity in kidney was significantly lower at all investigated time points, compared to other, metal-labeled, exendin-based tracers designed for PET and SPECT analyses [2, 3, 41]. Ex vivo autoradiography images (*blue inset*) show the decreasing [^18^F]exendin-4 accumulation in the kidney cortex at 60, 120, and 360 min p.i.

Measurements of [^18^F]exendin-4 in tissue samples indicated low but sustained uptake in the whole pancreas over the course of the study (Fig. 2a, Table 1). [^18^F]exendin-4 accumulation in the kidneys was high at first, but with time, its clearance was rapid and relatively little was retained (Fig. 3). A co-injection of excess exendin antagonist did not significantly reduce the renal uptake of [^18^F]exendin-4 (Table 1); this finding demonstrated that kidney tracer uptake was not mediated by the GLP-1R. In contrast, tracer uptake rates in the pancreas, lung, and stomach wall were significantly reduced after the exendin-3 injection, consistent with the expression profile of GLP-1R in rat [22].

**Table 1:**
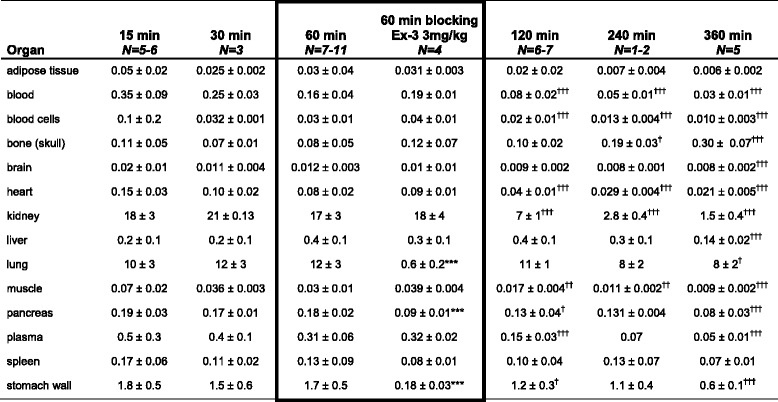
Ex vivo biodistribution of [^18^F]exendin-4 in rat

Differences between values at 60-min and 60-min blocking were determined with the Student *t* test without correction for multiple comparisons. One-way ANOVA and Bonferroni’s multiple comparison test was used to determine the differences at 60, 120, 240, and 360 min. */^†^ *P* < 0.05, **/^††^ *P* < 0.01, and ***/^†††^ *P* < 0.001 statistically significant compared to [^18^F]exendin-4 at 60 min post injection. Results are expressed as the mean ± SD percentage of the injected dose per gram of tissue

**Fig. 4:**
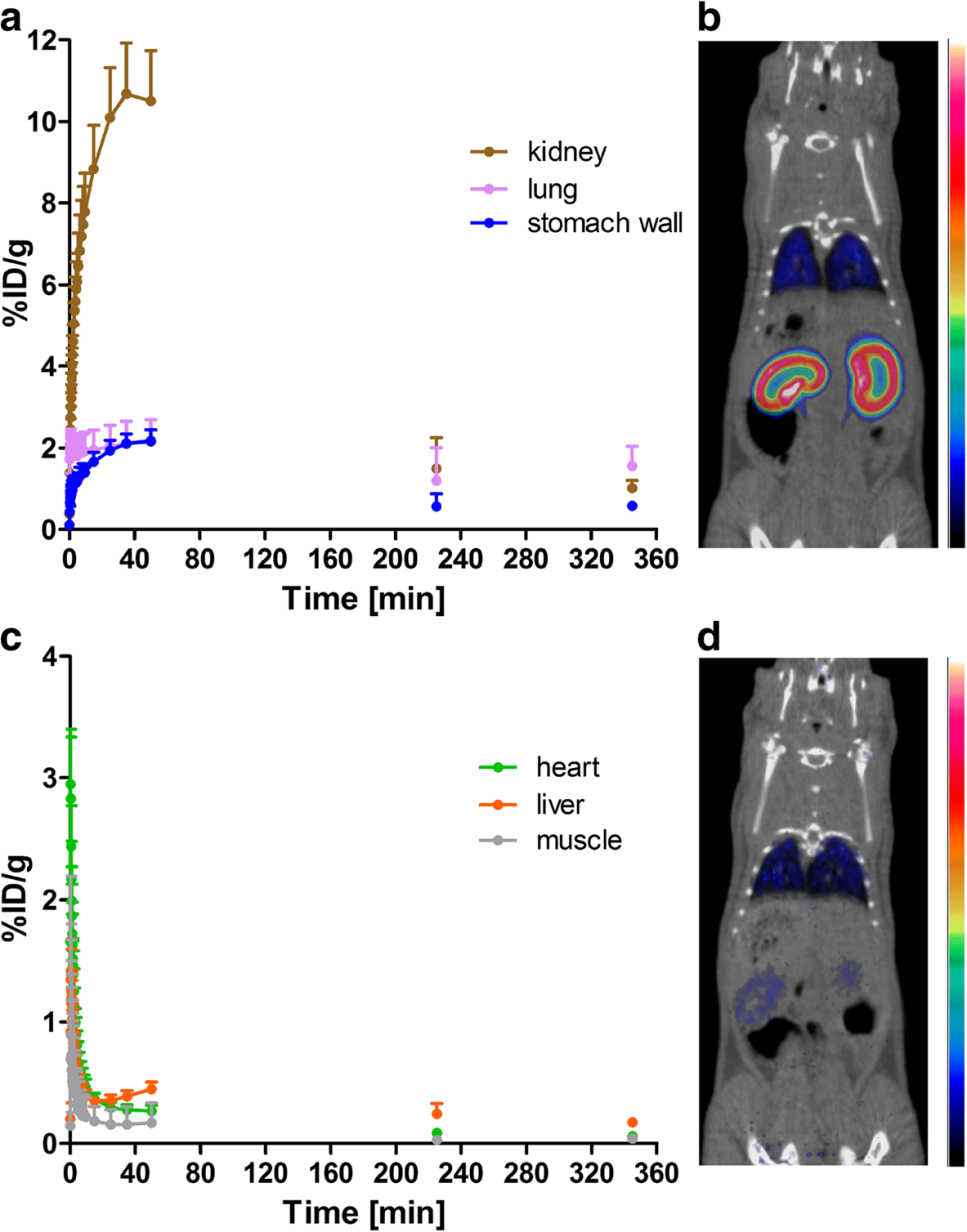
In vivo retention and clearance of [^18^F]exendin-4 in rat. **a** Dynamic PET imaging revealed rapid tracer accumulation in the kidneys at early time points and, thereafter, rapid elimination. Uptake in lung and stomach wall was modest. **b** Coronal PET/CT image at 0–60 min p.i. **c** Tracer accumulation in the liver remained low. **d** Coronal PET/CT image at 360 min p.i. (*N* = 2–5 rats/scan)

Dynamic imaging revealed increasing accumulation of [^18^F]exendin-4 in the kidneys within minutes p.i., and clearance had already begun in the late phase of the dynamic 60-min PET scan (Fig. 4a–b). Late static scans showed rapid elimination via the kidneys. The lung and stomach wall showed modest retention. Uptake in liver was low, which indicated that the tracer was not metabolized via the hepatic pathway (Fig. 4c–d). Both ex vivo biodistribution measurements and PET imaging showed very low uptake in the brain.

### Analysis of tracer metabolites

In rat plasma, we detected unchanged [^18^F]exendin-4 and a more polar radioactive metabolite(s). The plasma radioactivity associated with intact [^18^F]exendin-4 at 60 min p.i. represented 28.3 ± 3.5% of the total radioactivity, and at 120 min p.i., this level decreased to 16%. At 60 min p.i., [^18^F]exendin-4 represented 20 ± 7.2% of the radioactivity in the pancreas, compared to 38 ± 3.5% of the radioactivity in the duodenum, and negligible levels of intact [^18^F]exendin-4 in the kidney and urine. Additionally, in pigs, the plasma radioactivity associated with [^18^F]exendin-4 at 5 min p.i. represented 87 ± 6% of the radioactivity, and at 60 min p.i., this level decreased to 77 ± 3%.

### Effects of [^18^F]exendin-4 administration in pigs

In pigs, administration of [^18^F]exendin-4 raised the heart rate from 100–110 beats/min to 230–250 beats/min and body temperature from 37 to 41 °C. The blood pressure decreased, and arrhythmia was observed. Similar symptoms and effects of exendin on heart rate and blood pressure have been reported previously [23].

### Intrapancreatic distribution of [^18^F]exendin-4 in rats

**Fig. 5:**
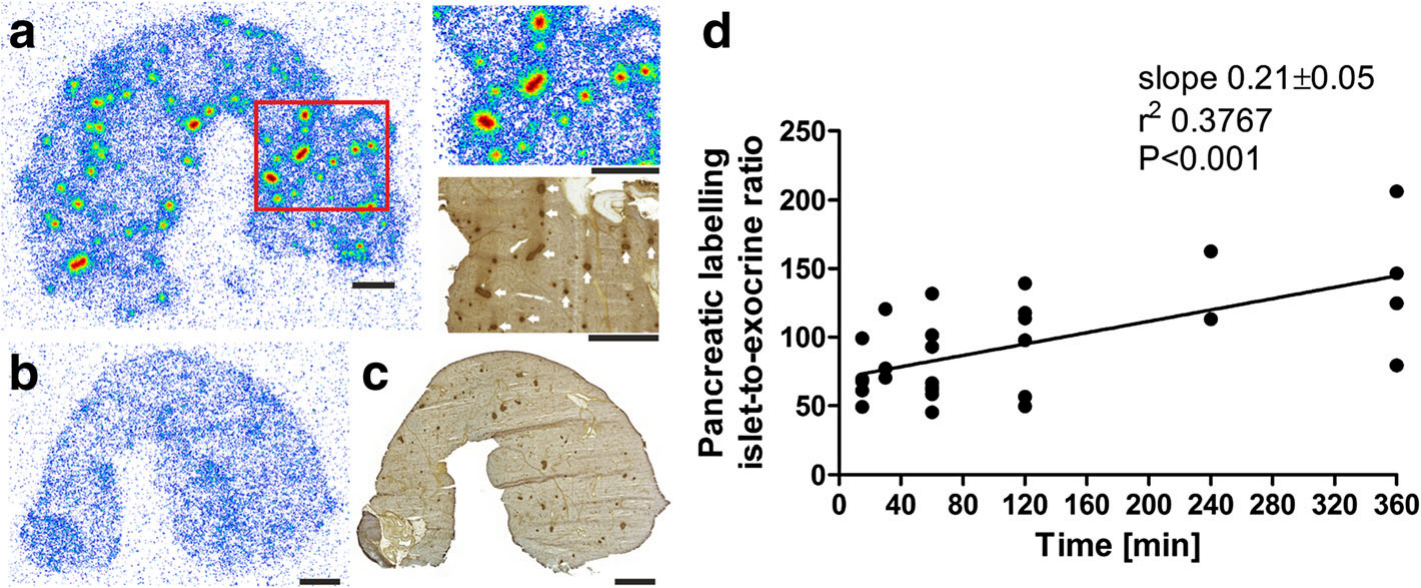
Ex vivo [^18^F]exendin-4 uptake in rat pancreas and pancreatic islets. **a** Tracer uptake in islets was clearly visible at 60 min p.i. Uptake in islets was verified by staining the same section with insulin antibody (*upper right inset* and *lower right inset*). **b**–**c** Tracer specificity was assessed by co-injecting [^18^F]exendin-4 and excess cold exendin-3. **d** Towards longer time points, the high ratios indicated a significant difference in islet labeling compared to background (*N* = 2–8 rats/time point). **a**–**c** Scale bars 2000 μm

After [^18^F]exendin-4 injections, tracer uptake was observed in the islets of rat pancreas (Fig. 5a). Islet labeling was not visible when rats were co-injected with excess cold exendin-3 and [^18^F]exendin-4 (Fig. 5b–c). These results indicated that [^18^F]exendin-4 had bound specifically to GLP-1R in the islets. The autoradiography results demonstrated that [^18^F]exendin-4 was cleared from the exocrine pancreas, but uptake was sustained in the islets. These findings were reflected in the islet-to-exocrine tissue ratio, which increased over time (Fig. 5d). These findings were consistent with previous reports [3, 8]. In contrast, our autoradiography results of rat brain did not show any tracer uptake in the investigated GLP1-R-rich areas after injections of either high (50 MBq) or low (5 MBq) doses of radioactivity.

**Fig. 6:**
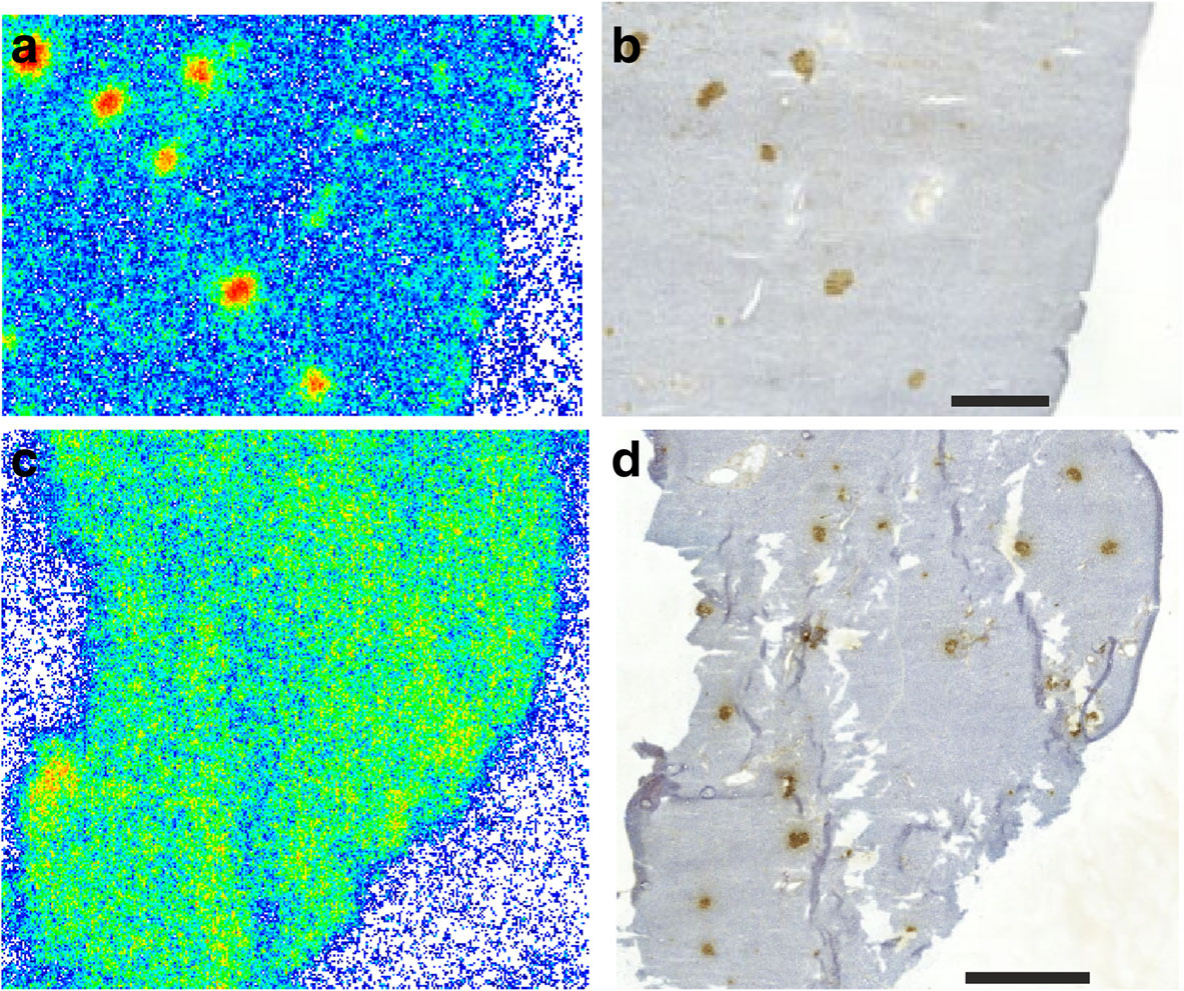
In vitro binding of [^18^F]exendin-4 in rat pancreas. **a** Clear tracer binding was observed after incubating pancreatic tissue sections with [^18^F]exendin-4 (2.5 nM) **b** co-localization with the islets was observed by staining the same sections with insulin antibody. **c**–**d** Islet labeling was not observed, when an excess of cold exendin-3 was added. Scale bar 1000 μm

**Fig. 7:**
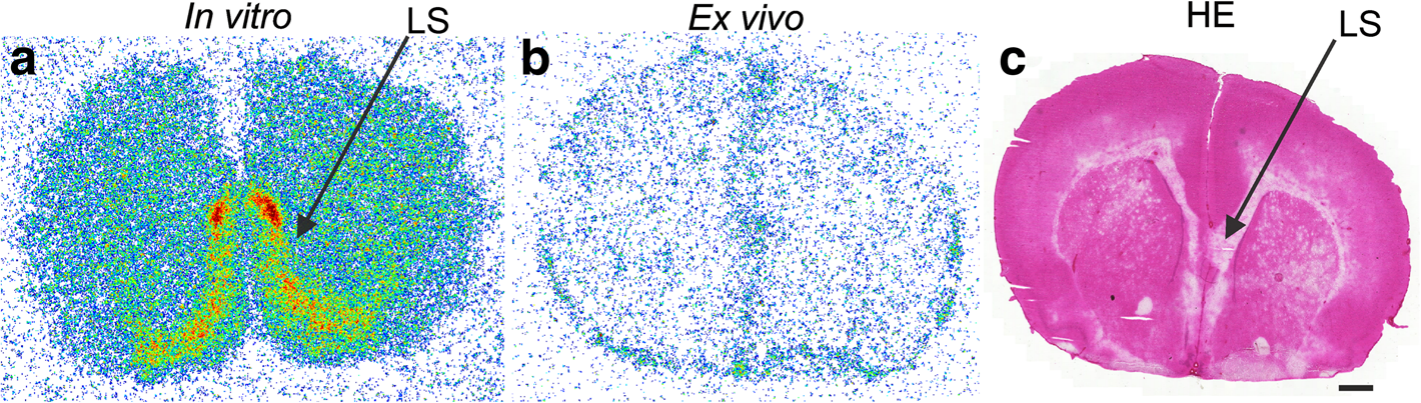
In vitro and ex vivo binding of [^18^F]exendin-4 in rat brain. **a** GLP-1R was clearly labeled in the *lateral septum* (LS) after incubating sections of rat brain with tracer in vitro (1.25 nM). **b** No binding in the corresponding brain area was observed 60 min after intravenous injection of [^18^F]exendin-4 (50 MBq). **c** For histological reference, the same section was stained with hematoxylin and eosin (HE). Scale bar 1000 μm

Similarly, islet labeling was observed in vitro, after incubating rat pancreatic sections with [^18^F]exendin-4 (Fig. 6a–b). Islet labeling was not detected when excess of cold exendin-3 was also added (Fig. 6c–d). Unlike the tracer injections into living animals, the GLP-1R areas in brain were clearly labeled after in vitro incubations with [^18^F]exendin-4 (Fig. 7).

### Tracer binding in tissue sections of human pancreas

**Fig. 8:**
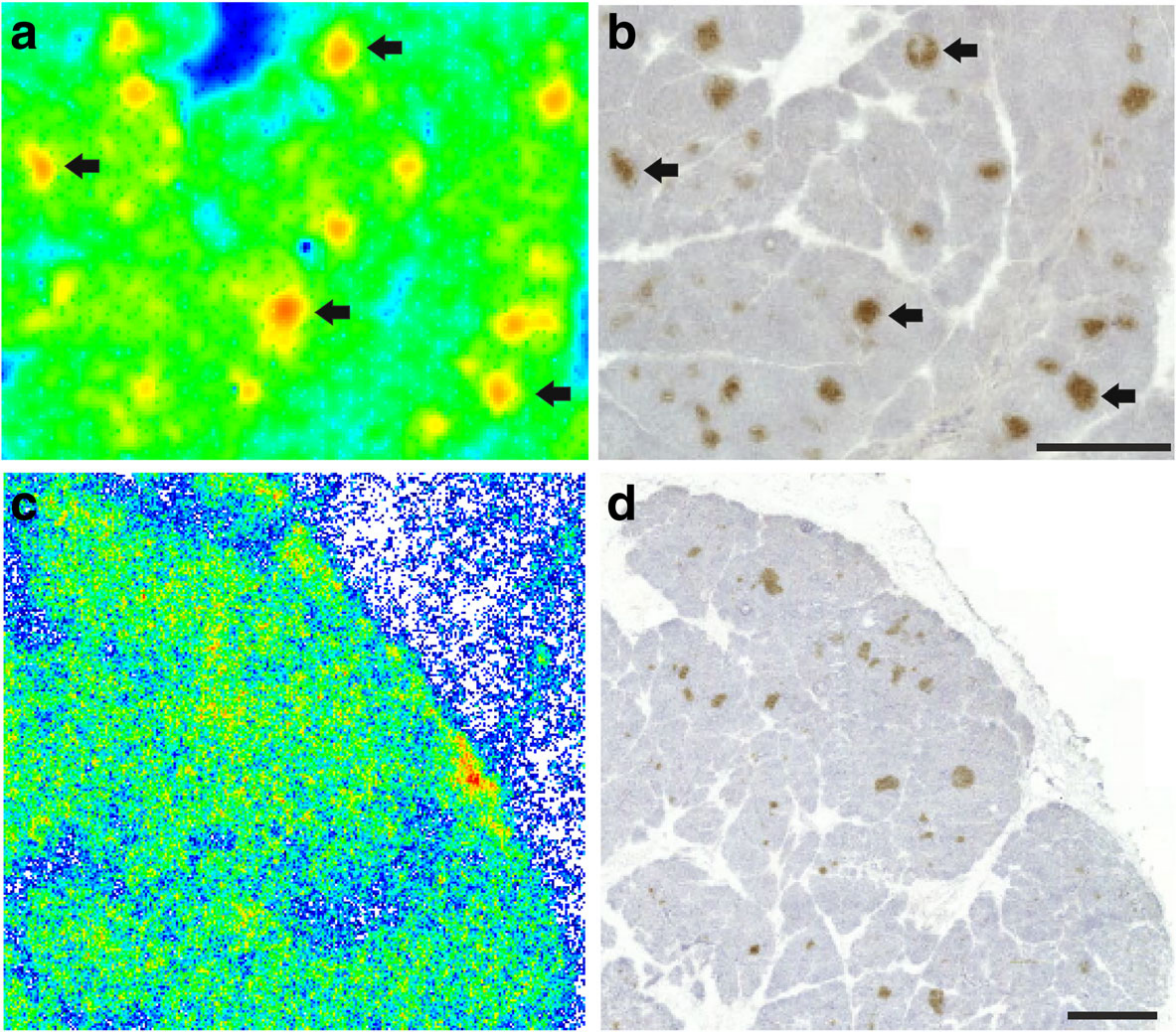
Islet labeling in human pancreas after [^18^F]exendin-4 incubation in vitro. **a**–**b** Insulin immunohistochemistry confirmed that tracer binding co-localized with the islets. **c**–**d** The specificity of [^18^F]exendin-4 to GLP-1R in the islets was demonstrated by incubating the sections with excess cold exendin-3. Scale bars 1000 μm

We investigated tracer binding in human pancreas by incubating pancreatic tissue sections with various concentrations of [^18^F]exendin-4. We found that tracer binding was clearly visible in human pancreatic islets (Fig. 8a–b). Islet binding was verified by immunostaining the same sections to detect insulin. When excess cold exendin-3 was added with [^18^F]exendin-4, no [^18^F]exendin-4 binding was observed in human pancreatic islet tissue sections (Fig. 8c–d).

### Dosimetry

By extrapolating from our rat ex vivo results, we calculated that the mean [^18^F]exendin-4 effective dose for a 70-kg human adult was 0.021 mSv/MBq (Table 2). The kidney was the dose-limiting organ. When we based calculations on the ex vivo results, the absorbed kidney dose for [^18^F]exendin-4 was 0.3 mSv/MBq.

**Table 2:** Human radiation dosimetry estimates for ^18^F radioactivity extrapolated from the rat ex vivo data

Organ	Dose (mSv/MBq)
Adrenal glands	0.016
Brain	0.004
Breasts	0.004
Gallbladder wall	0.011
LLI wall	0.003
Small intestine	0.005
Stomach wall	0.007
ULI wall	0.005
Heart wall	0.010
Kidneys	0.300
Liver	0.026
Lungs	0.071
Muscle	0.005
Ovaries	0.003
Pancreas	0.023
Red bone marrow	0.009
Osteogenic cells	0.045
Skin	0.003
Spleen	0.022
Testes	0.001
Thymus	0.005
Thyroid	0.003
Urinary bladder wall	0.002
Uterus	0.003
Total body	0.009
Effective dose	0.021

*LLI* lower large intestine wall, *ULI* upper large intestine

## Discussion

This report described the synthesis of [^18^F]exendin-4 and its evaluation in rat and human pancreas. In this study, ex vivo γ-counting of rat whole pancreas and autoradiography analyses of pancreatic sections showed specific and sustained uptake of [^18^F]exendin-4 in the islets. In vitro labeling of human pancreas revealed specific targeting of the islets and co-localization with insulin, detected with immunohistochemistry. Renal clearance of [^18^F]exendin-4 was rapid compared to radiometal-labeled exendin derivatives [2, 3, 24, 25].

^18^F-labeled target-specific peptides are ideal in vivo imaging agents, because ^18^F is readily available from most small medical cyclotrons, and it has a half-life (110 min) that matches the pharmacokinetics of most peptides. Moreover, ^18^F decays with a 96% positron branching ratio, and it has a short β^+^ trajectory (*E*_βmax_ = 635 keV), which enables the acquisition of high-resolution PET images. Previous reports have described various approaches for the radiofluorination of exendin-4 analogs, which utilize maleimide- or aldehyde-containing prosthetic reagents, and chelating agents [13, 26–28]. However, the main challenge is designing an efficient strategy for preparing radiotracers with very high specific activity, which would render in vivo imaging of native β cells. Click chemistry has generated increasing attention due to its rapid and high-yielding transformation, and tolerance to a broad range of functional groups. These properties make click chemistry one of the preferred strategies in developing ^18^F-labeled radiopharmaceuticals [14, 29, 30]. The original copper-catalyzed variant of the Huisgen 1,3-dipolar cycloaddition of terminal alkynes and azides features very high reaction rates [31, 32] and has prompted us to advance this reaction into the design of a potential clinical grade tracer for native β cell imaging. It was shown previously that the binding affinity (IC_50_) of native exendin-4 for GLP-1 receptor was not significantly altered by modification/addition of a fluorinated linker [14, 33]. Noteworthy, the different fluorination strategies did not affect the binding affinity of the cold ligand towards the target receptor, compared to native exendin-4.

The GLP-1R is expressed specifically in pancreatic β cells [34, 35]; little to no expression was found on other pancreatic islet cell types [36]. Because GLP-1R expression levels are low, a sensitive radiotracer with high specific activity is required to avoid receptor saturation with unlabeled peptide [37]. The number of GLP-1Rs in native β cells and transplanted islets is restricted compared to insulinomas, which overexpress the receptor. In this study, [^18^F]exendin-4 was produced with a specific activity as high as 323 MBq/nmol and an injected mass of 2 ± 1 μg/kg. In humans, the pharmacological dose of exenatide (Byetta®) is low (10 μg BID), and in animal studies, exendin-4 was a potent glucose-lowering agent (dose 0.001 to 10 μg) [38]. These properties of high specific activity and low injected radiotracer mass must be considered carefully. With doses of 7–25 μg of [^68^Ga]NOTA-exendin-4, subjects experienced palpitation, vomiting, and nausea after tracer injection [39].

In previous studies, the levels of pancreatic uptake of other labeled exendin-based tracers were similar at 60 min p.i. (Fig. 2b). Nevertheless, [^18^F]exendin-4 accumulation in the pancreas was significantly higher than that of the ^64^Cu- and ^68^Ga-labeled counterparts [3]. Brom et al. [2] showed that the [^111^In]DTPA-exendin-4 SPECT tracer was taken up by the pancreas at a higher rate than the PET ligands.

Kastin et al. [40] reported that exendin-4 crossed the blood–brain barrier in rat. In our studies, GLP-1R-rich areas were observed in in vitro studies of rat brain sections (Fig. 7). However, ex vivo autoradiography, tissue γ-counting, and PET imaging did not reveal any specific binding in rat brain. These results indicated that [^18^F]exendin-4 did not cross the blood–brain barrier.

Our stability study results demonstrated that the native (unmetabolized) tracer was recovered in significantly higher amounts in pig plasma compared to rat plasma. However, in rats [^18^F]exendin-4 was rapidly cleared from the blood, and low amounts were taken up in the liver. In contrast, when ^18^F-labeled GLP-1R targeting radiotracers were investigated in xenograft models, substantial liver uptake was observed [11, 13, 14, 28]. On the other hand, Yue et al. [12] reported moderate liver retention of [^18^F]FNEM-[Cys^40^]-exendin-4 in a xenograft model. High liver uptake might restrict the clinical use of these tracers, because the liver is the site of insulinoma metastasis, and it is also the primary site for islet transplantation. Low tracer accumulation in the liver enables the high target-to-background contrast required for high-quality PET imaging.

In this study, PET imaging and organ radioactivity measurements demonstrated high [^18^F]exendin-4 uptake in the kidneys soon after injection, but thereafter, clearance was rapid and retention decreased (Fig. 3). In contrast, the renal excretion route and proximal tubular reabsorption cause high retention of metal-labeled exendin tracers in the kidney cortex. At the 60-min time point, kidney uptake of [^18^F]exendin-4 was significantly lower than the uptake of the metal-labeled counterparts. Furthermore, at several hours p.i., high levels of both [^111^In]DTPA-exendin-4 [2, 41] and [^64^Cu]NODAGA-exendin-4 [3] had accumulated at in the kidneys, and [^111^In]DTPA-exendin-4 showed only minor clearance over time. Consequently, repeated scanning in patients could cause radiation doses that exceed the tolerance of this delicate organ [4, 42]. Melis et al. [43] showed that high radiation doses of [^111^In]DTPA-Lys^40^-exendin-4 caused nephrotoxicity in mice. Several approaches have been tested to overcome high uptake of exendin-based tracers in the kidneys, including co-administration of competitive inhibitors of reabsorption [5–7], addition of metabolizable linkers [8, 9], or modifications in the size, charge, or structure of the tracer [44, 45]. Those attempts have been crucial in finding ways to improve the imaging accuracy, decrease the risk of nephrotoxicity, and improve the efficacy of peptide receptor radionuclide therapy.

In this study, the absorbed kidney dose of [^18^F]exendin-4 was similar to that of [^68^Ga]Ga-DO3A-VS-Cys^40^-exendin-4 (Table 3) [46]. The absorbed kidney doses of ^64^Cu- and ^111^In-labeled tracers were 5 and 15 times higher, respectively, than that of [^18^F]exendin-4. Furthermore, the effective dose of [^18^F]exendin-4 was 4 and 7 times lower than corresponding values for [^64^Cu]NODAGA-exendin-4 and [^111^In]DTPA-exendin-4.

**Table 3:** Absorbed kidney dose and effective dose of ^18^F-labeled tracer and metal-labeled exendin PET and SPECT tracers

Radiotracer	Kidney dose [mSv/MBq]	Effective dose [mSv/MBq]
[^18^F]exendin-4	0.300	0.021
[^68^Ga]Ga-DO3A-VS-Cys^40^-exendin-4 [46]	0.276	0.016
[^64^Cu]NODAGA-exendin-4 [3]	1.48	0.074
[^111^In]DTPA-exendin-4 [7]	4.48	0.155

## Conclusions

We found that in vivo injections of the β cell-specific PET tracer, [^18^F]exendin-4, resulted in specific, sustained tracer uptake in rat pancreatic islets. Additionally, we demonstrated specific uptake of [^18^F]exendin-4 in human pancreatic tissues in vitro. Unlike its metal-labeled counterparts, [^18^F]exendin-4 renal clearance was rapid. These properties of [^18^F]exendin-4 showed promise for its future development for use in clinical imaging of β cells.

## Abbreviations

[^18^F]exendin-4: ^18^F-labeled [Nle^14^,Lys^40^]exendin-4
BID: Bis in die
BSA: Bovine serum albumin
CT: Computed tomography
DMSO: Dimethyl sulfoxide
GLP-1: Glucagon-like peptide-1
GLP-1R: Glucagon-like peptide-1 receptor
HPLC: High-performance liquid chromatography
i.v.: Intravenous
p.i.: Post injection
PBS: Phosphate-buffered saline
PET: Positron emission tomography
PSL: Photostimulated luminescence
TLC: Thin layer chromatography
VOI: Volume of interest
